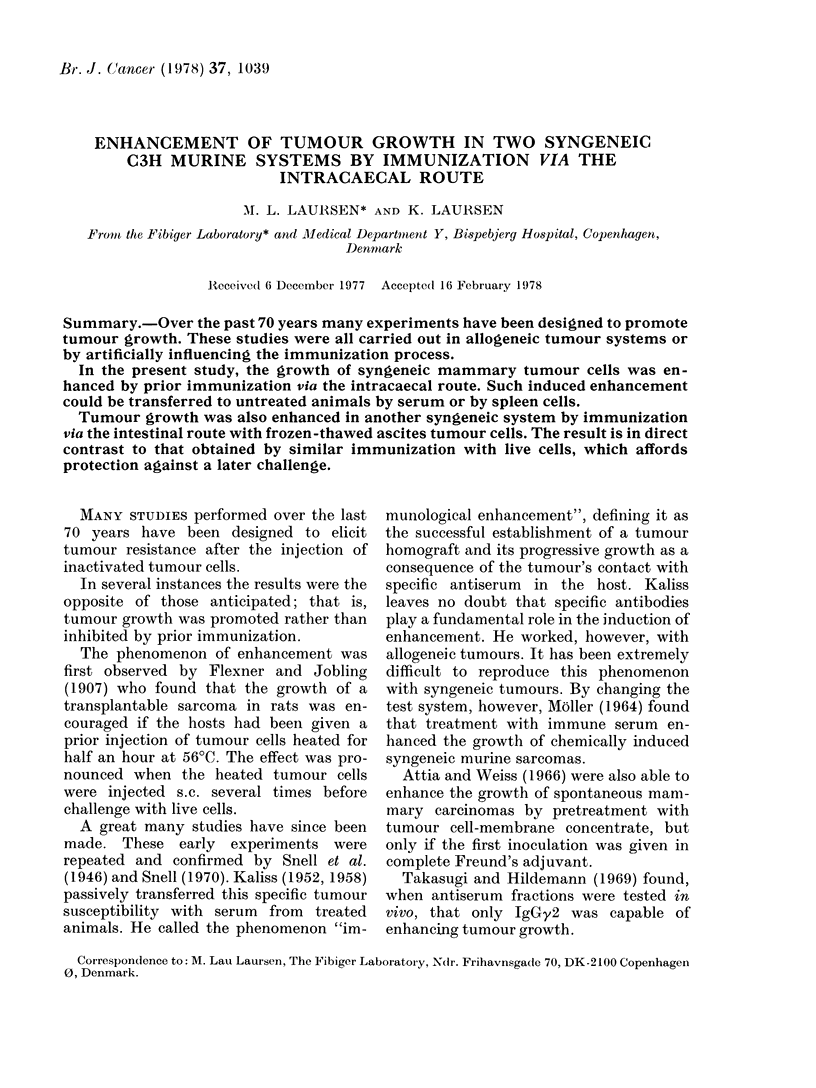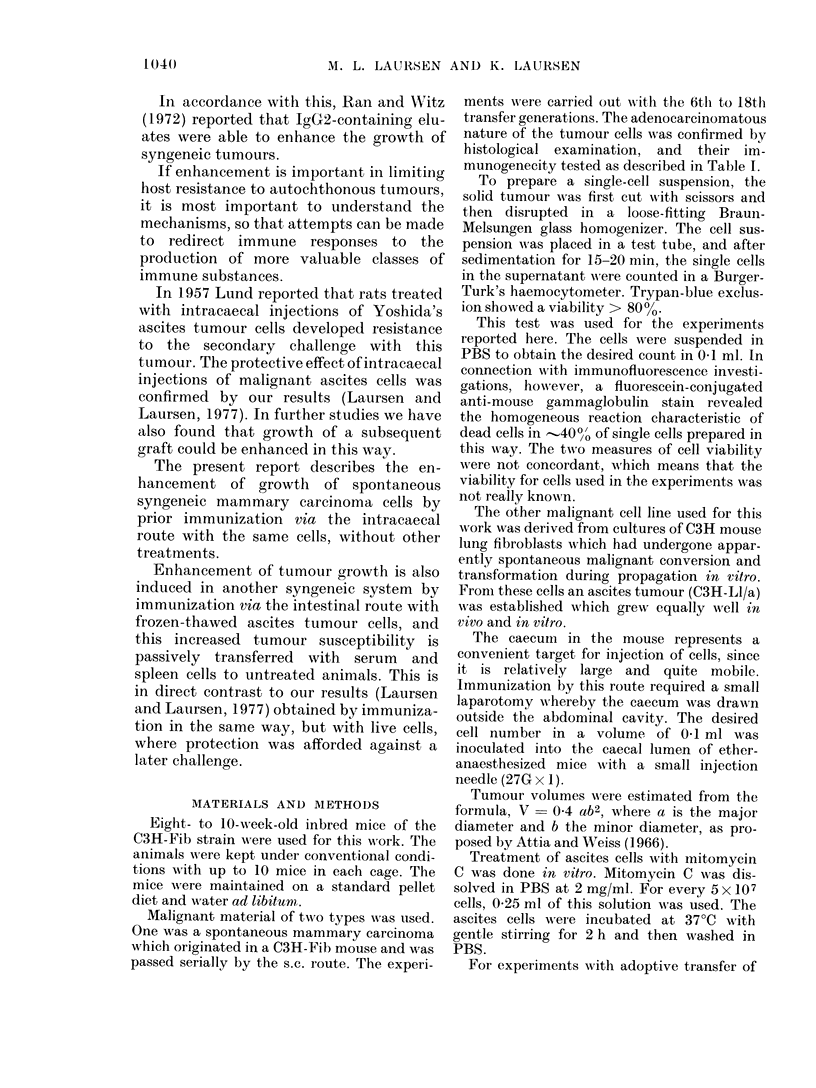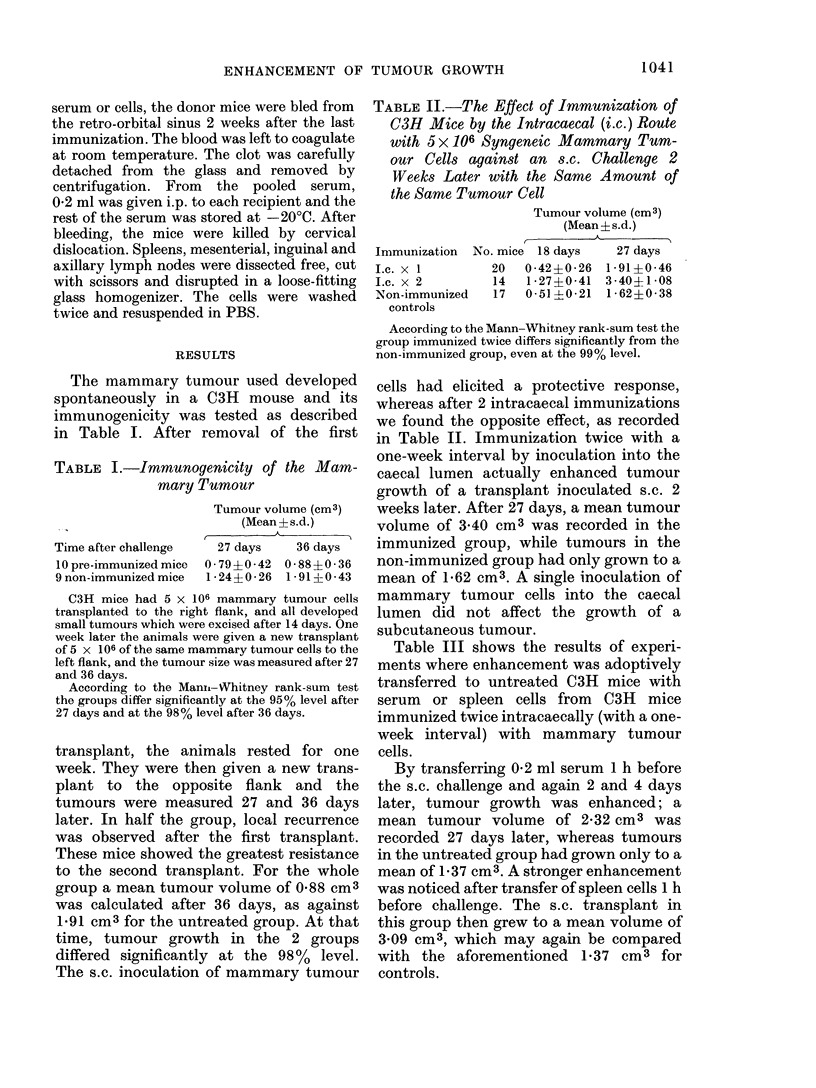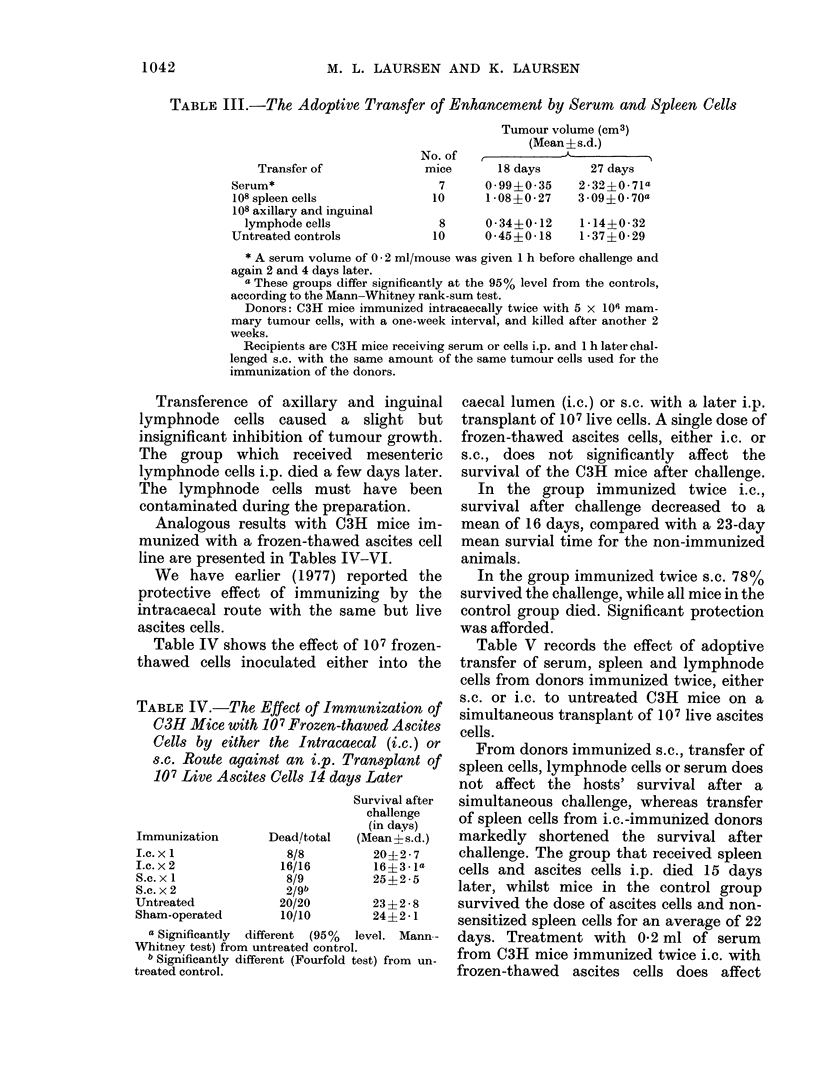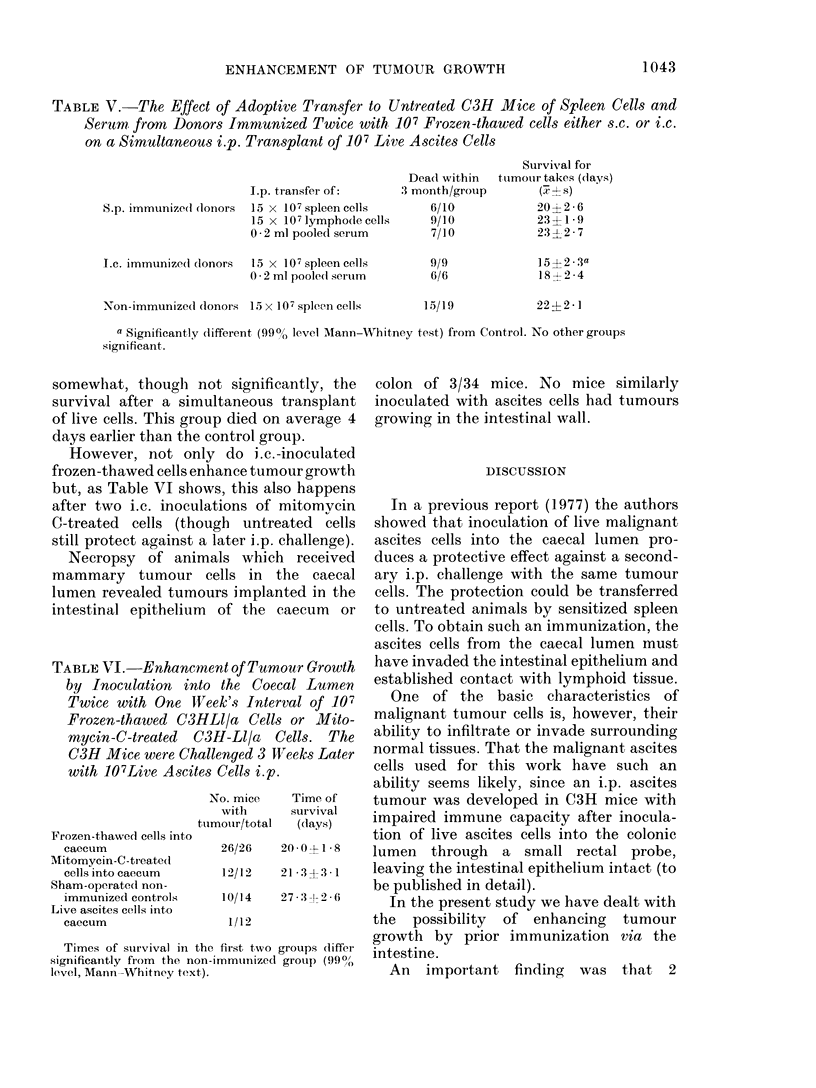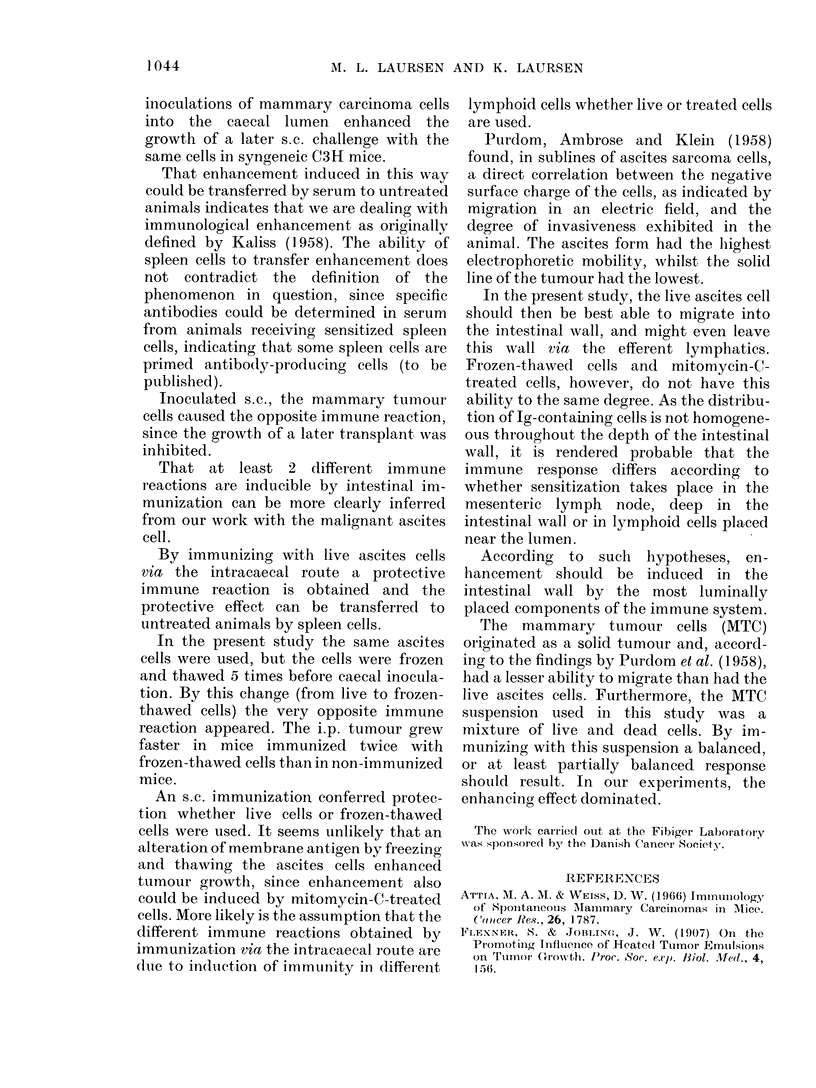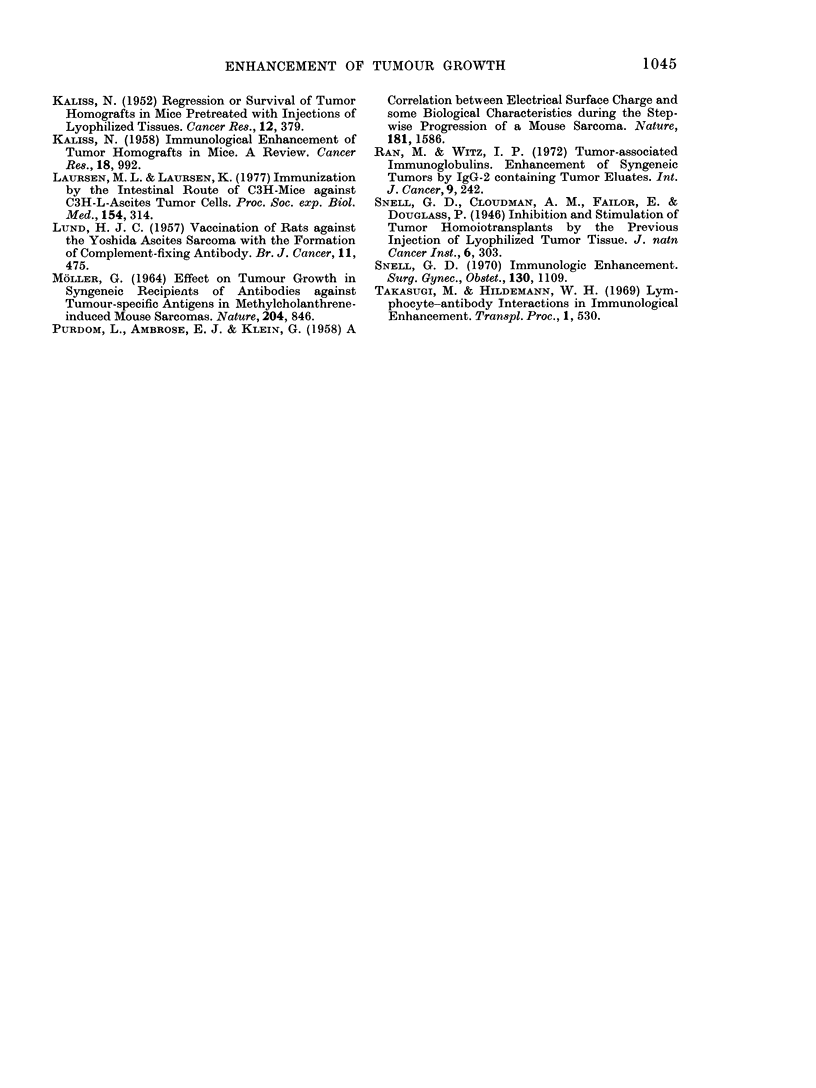# Enhancement of tumour growth in two syngeneic C3H murine systems by immunization via the intracaecal route.

**DOI:** 10.1038/bjc.1978.151

**Published:** 1978-06

**Authors:** M. L. Laursen, K. Laursen

## Abstract

Over the past 70 years many experiments have been designed to promote tumour growth. These studies were all carried out in allogeneic tumour systems or by artificially influencing the immunization process. In the present study, the growth of syngeneic mammary tumour cells was enhanced by prior immunization via the intracaecal route. Such induced enhancement could be transferred to untreated animals by serum or by spleen cells. Tumour growth was also enhanced in another syngeneic system by immunization via the intestinal route with frozen-thawed ascites tumour cells. The result is in direct contrast to that obtained by similar immunization with live cells, which affords protection against a later challenge.


*Br. J. Cancer* (1978) **37**, 1039

### ENHANCEMENT OF TUMOUR GROWTH IN TWO SYNGENEIC C3H MURINE SYSTEMS BY IMMUNIZATION *VIA* THE INTRACAECAL ROUTE

M. L. LAURSEN* AND K. LAURSEN

*From the Fibiger Laboratory* and Medical Department Y, Bispebjerg Hospital, Copenhagen, Denmark*

Received 6 December 1977 Accepted 16 February 1978

**Summary.—Over the past 70 years many experiments have been designed to promote tumour growth. These studies were all carried out in allogeneic tumour systems or by artificially influencing the immunization process.**

**In the present study, the growth of syngeneic mammary tumour cells was enhanced by prior immunization *via* the intracaecal route. Such induced enhancement could be transferred to untreated animals by serum or by spleen cells.**

**Tumour growth was also enhanced in another syngeneic system by immunization *via* the intestinal route with frozen-thawed ascites tumour cells. The result is in direct contrast to that obtained by similar immunization with live cells, which affords protection against a later challenge.**

MANY STUDIES performed over the last 70 years have been designed to elicit tumour resistance after the injection of inactivated tumour cells.

In several instances the results were the opposite of those anticipated; that is, tumour growth was promoted rather than inhibited by prior immunization.

The phenomenon of enhancement was first observed by Flexner and Jobling (1907) who found that the growth of a transplantable sarcoma in rats was encouraged if the hosts had been given a prior injection of tumour cells heated for half an hour at 56°C. The effect was pronounced when the heated tumour cells were injected s.c. several times before challenge with live cells.

A great many studies have since been made. These early experiments were repeated and confirmed by Snell *et al.* (1946) and Snell (1970). Kaliss (1952, 1958) passively transferred this specific tumour susceptibility with serum from treated animals. He called the phenomenon "immunological enhancement", defining it as the successful establishment of a tumour homograft and its progressive growth as a consequence of the tumour's contact with specific antiserum in the host. Kaliss leaves no doubt that specific antibodies play a fundamental role in the induction of enhancement. He worked, however, with allogeneic tumours. It has been extremely difficult to reproduce this phenomenon with syngeneic tumours. By changing the test system, however, Möller (1964) found that treatment with immune serum enhanced the growth of chemically induced syngeneic murine sarcomas.

Attia and Weiss (1966) were also able to enhance the growth of spontaneous mammary carcinomas by pretreatment with tumour cell-membrane concentrate, but only if the first inoculation was given in complete Freund's adjuvant.

Takasugi and Hildemann (1969) found, when antiserum fractions were tested *in vivo*, that only IgG$\gamma$2 was capable of enhancing tumour growth.

Correspondence to: M. Lau Laursen, The Fibiger Laboratory, Ndr. Frihavnsgade 70, DK-2100 Copenhagen Ø, Denmark.

In accordance with this, Ran and Witz (1972) reported that IgG2-containing eluates were able to enhance the growth of syngeneic tumours.

If enhancement is important in limiting host resistance to autochthonous tumours, it is most important to understand the mechanisms, so that attempts can be made to redirect immune responses to the production of more valuable classes of immune substances.

In 1957 Lund reported that rats treated with intracaecal injections of Yoshida's ascites tumour cells developed resistance to the secondary challenge with this tumour. The protective effect of intracaecal injections of malignant ascites cells was confirmed by our results (Laursen and Laursen, 1977). In further studies we have also found that growth of a subsequent graft could be enhanced in this way.

The present report describes the enhancement of growth of spontaneous syngeneic mammary carcinoma cells by prior immunization *via* the intracaecal route with the same cells, without other treatments.

Enhancement of tumour growth is also induced in another syngeneic system by immunization *via* the intestinal route with frozen-thawed ascites tumour cells, and this increased tumour susceptibility is passively transferred with serum and spleen cells to untreated animals. This is in direct contrast to our results (Laursen and Laursen, 1977) obtained by immunization in the same way, but with live cells, where protection was afforded against a later challenge.

### MATERIALS AND METHODS

Eight- to 10-week-old inbred mice of the C3H-Fib strain were used for this work. The animals were kept under conventional conditions with up to 10 mice in each cage. The mice were maintained on a standard pellet diet and water *ad libitum.*

Malignant material of two types was used. One was a spontaneous mammary carcinoma which originated in a C3H-Fib mouse and was passed serially by the s.c. route. The experiments were carried out with the 6th to 18th transfer generations. The adenocarcinomatous nature of the tumour cells was confirmed by histological examination, and their immunogenecity tested as described in Table I.

To prepare a single-cell suspension, the solid tumour was first cut with scissors and then disrupted in a loose-fitting Braun-Melsungen glass homogenizer. The cell suspension was placed in a test tube, and after sedimentation for 15–20 min, the single cells in the supernatant were counted in a Burger-Turk's haemocytometer. Trypan-blue exclusion showed a viability $> 80\%$.

This test was used for the experiments reported here. The cells were suspended in PBS to obtain the desired count in 0·1 ml. In connection with immunofluorescence investigations, however, a fluorescein-conjugated anti-mouse gammaglobulin stain revealed the homogeneous reaction characteristic of dead cells in $\sim 40\%$ of single cells prepared in this way. The two measures of cell viability were not concordant, which means that the viability for cells used in the experiments was not really known.

The other malignant cell line used for this work was derived from cultures of C3H mouse lung fibroblasts which had undergone apparently spontaneous malignant conversion and transformation during propagation *in vitro.* From these cells an ascites tumour (C3H-Ll/a) was established which grew equally well *in vivo* and *in vitro.*

The caecum in the mouse represents a convenient target for injection of cells, since it is relatively large and quite mobile. Immunization by this route required a small laparotomy whereby the caecum was drawn outside the abdominal cavity. The desired cell number in a volume of 0·1 ml was inoculated into the caecal lumen of ether-anaesthesized mice with a small injection needle ($27G \times 1$).

Tumour volumes were estimated from the formula, $V = 0{\cdot}4\ ab^2$, where $a$ is the major diameter and $b$ the minor diameter, as proposed by Attia and Weiss (1966).

Treatment of ascites cells with mitomycin C was done *in vitro.* Mitomycin C was dissolved in PBS at 2 mg/ml. For every $5 \times 10^7$ cells, 0·25 ml of this solution was used. The ascites cells were incubated at 37°C with gentle stirring for 2 h and then washed in PBS.

For experiments with adoptive transfer of serum or cells, the donor mice were bled from the retro-orbital sinus 2 weeks after the last immunization. The blood was left to coagulate at room temperature. The clot was carefully detached from the glass and removed by centrifugation. From the pooled serum, 0·2 ml was given i.p. to each recipient and the rest of the serum was stored at −20°C. After bleeding, the mice were killed by cervical dislocation. Spleens, mesenterial, inguinal and axillary lymph nodes were dissected free, cut with scissors and disrupted in a loose-fitting glass homogenizer. The cells were washed twice and resuspended in PBS.

### RESULTS

The mammary tumour used developed spontaneously in a C3H mouse and its immunogenicity was tested as described in Table I. After removal of the first transplant, the animals rested for one week. They were then given a new transplant to the opposite flank and the tumours were measured 27 and 36 days later. In half the group, local recurrence was observed after the first transplant. These mice showed the greatest resistance to the second transplant. For the whole group a mean tumour volume of 0·88 $cm^3$ was calculated after 36 days, as against 1·91 $cm^3$ for the untreated group. At that time, tumour growth in the 2 groups differed significantly at the 98% level. The s.c. inoculation of mammary tumour cells had elicited a protective response, whereas after 2 intracaecal immunizations we found the opposite effect, as recorded in Table II. Immunization twice with a one-week interval by inoculation into the caecal lumen actually enhanced tumour growth of a transplant inoculated s.c. 2 weeks later. After 27 days, a mean tumour volume of 3·40 $cm^3$ was recorded in the immunized group, while tumours in the non-immunized group had only grown to a mean of 1·62 $cm^3$. A single inoculation of mammary tumour cells into the caecal lumen did not affect the growth of a subcutaneous tumour.

TABLE I.—*Immunogenicity of the Mammary Tumour*

| | Tumour volume ($cm^3$) (Mean ± s.d.) | |
|---|---|---|
| Time after challenge | 27 days | 36 days |
| 10 pre-immunized mice | 0·79 ± 0·42 | 0·88 ± 0·36 |
| 9 non-immunized mice | 1·24 ± 0·26 | 1·91 ± 0·43 |

C3H mice had 5 × $10^6$ mammary tumour cells transplanted to the right flank, and all developed small tumours which were excised after 14 days. One week later the animals were given a new transplant of 5 × $10^6$ of the same mammary tumour cells to the left flank, and the tumour size was measured after 27 and 36 days.

According to the Mann–Whitney rank-sum test the groups differ significantly at the 95% level after 27 days and at the 98% level after 36 days.

TABLE II.—*The Effect of Immunization of C3H Mice by the Intracaecal (i.c.) Route with 5 × $10^6$ Syngeneic Mammary Tumour Cells against an s.c. Challenge 2 Weeks Later with the Same Amount of the Same Tumour Cell*

| | | Tumour volume ($cm^3$) (Mean ± s.d.) | |
|---|---|---|---|
| Immunization | No. mice | 18 days | 27 days |
| I.c. × 1 | 20 | 0·42 ± 0·26 | 1·91 ± 0·46 |
| I.c. × 2 | 14 | 1·27 ± 0·41 | 3·40 ± 1·08 |
| Non-immunized controls | 17 | 0·51 ± 0·21 | 1·62 ± 0·38 |

According to the Mann–Whitney rank-sum test the group immunized twice differs significantly from the non-immunized group, even at the 99% level.

Table III shows the results of experiments where enhancement was adoptively transferred to untreated C3H mice with serum or spleen cells from C3H mice immunized twice intracaecally (with a one-week interval) with mammary tumour cells.

By transferring 0·2 ml serum 1 h before the s.c. challenge and again 2 and 4 days later, tumour growth was enhanced; a mean tumour volume of 2·32 $cm^3$ was recorded 27 days later, whereas tumours in the untreated group had grown only to a mean of 1·37 $cm^3$. A stronger enhancement was noticed after transfer of spleen cells 1 h before challenge. The s.c. transplant in this group then grew to a mean volume of 3·09 $cm^3$, which may again be compared with the aforementioned 1·37 $cm^3$ for controls.

TABLE III.—*The Adoptive Transfer of Enhancement by Serum and Spleen Cells*

| Transfer of | No. of mice | Tumour volume (cm³) (Mean ± s.d.) 18 days | 27 days |
|---|---|---|---|
| Serum* | 7 | 0·99±0·35 | 2·32±0·71[a] |
| $10^8$ spleen cells | 10 | 1·08±0·27 | 3·09±0·70[a] |
| $10^8$ axillary and inguinal lymphode cells | 8 | 0·34±0·12 | 1·14±0·32 |
| Untreated controls | 10 | 0·45±0·18 | 1·37±0·29 |

* A serum volume of 0·2 ml/mouse was given 1 h before challenge and again 2 and 4 days later.

[a] These groups differ significantly at the 95% level from the controls, according to the Mann–Whitney rank-sum test.

Donors: C3H mice immunized intracaecally twice with $5 \times 10^6$ mammary tumour cells, with a one-week interval, and killed after another 2 weeks.

Recipients are C3H mice receiving serum or cells i.p. and 1 h later challenged s.c. with the same amount of the same tumour cells used for the immunization of the donors.

Transference of axillary and inguinal lymphnode cells caused a slight but insignificant inhibition of tumour growth. The group which received mesenteric lymphnode cells i.p. died a few days later. The lymphnode cells must have been contaminated during the preparation.

Analogous results with C3H mice immunized with a frozen-thawed ascites cell line are presented in Tables IV–VI.

We have earlier (1977) reported the protective effect of immunizing by the intracaecal route with the same but live ascites cells.

Table IV shows the effect of $10^7$ frozen-thawed cells inoculated either into the caecal lumen (i.c.) or s.c. with a later i.p. transplant of $10^7$ live cells. A single dose of frozen-thawed ascites cells, either i.c. or s.c., does not significantly affect the survival of the C3H mice after challenge.

TABLE IV.—*The Effect of Immunization of C3H Mice with $10^7$ Frozen-thawed Ascites Cells by either the Intracaecal (i.c.) or s.c. Route against an i.p. Transplant of $10^7$ Live Ascites Cells 14 days Later*

| Immunization | Dead/total | Survival after challenge (in days) (Mean ± s.d.) |
|---|---|---|
| I.c. × 1 | 8/8 | 20±2·7 |
| I.c. × 2 | 16/16 | 16±3·1[a] |
| S.c. × 1 | 8/9 | 25±2·5 |
| S.c. × 2 | 2/9[b] | |
| Untreated | 20/20 | 23±2·8 |
| Sham-operated | 10/10 | 24±2·1 |

[a] Significantly different (95% level. Mann-Whitney test) from untreated control.

[b] Significantly different (Fourfold test) from untreated control.

In the group immunized twice i.c., survival after challenge decreased to a mean of 16 days, compared with a 23-day mean survial time for the non-immunized animals.

In the group immunized twice s.c. 78% survived the challenge, while all mice in the control group died. Significant protection was afforded.

Table V records the effect of adoptive transfer of serum, spleen and lymphnode cells from donors immunized twice, either s.c. or i.c. to untreated C3H mice on a simultaneous transplant of $10^7$ live ascites cells.

From donors immunized s.c., transfer of spleen cells, lymphnode cells or serum does not affect the hosts' survival after a simultaneous challenge, whereas transfer of spleen cells from i.c.-immunized donors markedly shortened the survival after challenge. The group that received spleen cells and ascites cells i.p. died 15 days later, whilst mice in the control group survived the dose of ascites cells and non-sensitized spleen cells for an average of 22 days. Treatment with 0·2 ml of serum from C3H mice immunized twice i.c. with frozen-thawed ascites cells does affect

TABLE V.—*The Effect of Adoptive Transfer to Untreated C3H Mice of Spleen Cells and Serum from Donors Immunized Twice with $10^7$ Frozen-thawed cells either s.c. or i.c. on a Simultaneous i.p. Transplant of $10^7$ Live Ascites Cells*

| | I.p. transfer of: | Dead within 3 month/group | Survival for tumour takes (days) ($\bar{x} \pm s$) |
|---|---|---|---|
| S.p. immunized donors | $15 \times 10^7$ spleen cells | 6/10 | $20 \pm 2 \cdot 6$ |
| | $15 \times 10^7$ lymphode cells | 9/10 | $23 \pm 1 \cdot 9$ |
| | 0·2 ml pooled serum | 7/10 | $23 \pm 2 \cdot 7$ |
| I.c. immunized donors | $15 \times 10^7$ spleen cells | 9/9 | $15 \pm 2 \cdot 3$[a] |
| | 0·2 ml pooled serum | 6/6 | $18 \pm 2 \cdot 4$ |
| Non-immunized donors | $15 \times 10^7$ spleen cells | 15/19 | $22 \pm 2 \cdot 1$ |

[a] Significantly different (99% level Mann–Whitney test) from Control. No other groups significant.

somewhat, though not significantly, the survival after a simultaneous transplant of live cells. This group died on average 4 days earlier than the control group.

However, not only do i.c.-inoculated frozen-thawed cells enhance tumour growth but, as Table VI shows, this also happens after two i.c. inoculations of mitomycin C-treated cells (though untreated cells still protect against a later i.p. challenge).

Necropsy of animals which received mammary tumour cells in the caecal lumen revealed tumours implanted in the intestinal epithelium of the caecum or colon of 3/34 mice. No mice similarly inoculated with ascites cells had tumours growing in the intestinal wall.

TABLE VI.—*Enhancment of Tumour Growth by Inoculation into the Coecal Lumen Twice with One Week's Interval of $10^7$ Frozen-thawed C3HLl/a Cells or Mitomycin-C-treated C3H-Ll/a Cells. The C3H Mice were Challenged 3 Weeks Later with $10^7$Live Ascites Cells i.p.*

| | No. mice with tumour/total | Time of survival (days) |
|---|---|---|
| Frozen-thawed cells into caecum | 26/26 | $20 \cdot 0 \pm 1 \cdot 8$ |
| Mitomycin-C-treated cells into caecum | 12/12 | $21 \cdot 3 \pm 3 \cdot 1$ |
| Sham-operated non-immunized controls | 10/14 | $27 \cdot 3 \pm 2 \cdot 6$ |
| Live ascites cells into caecum | 1/12 | |

Times of survival in the first two groups differ significantly from the non-immunized group (99% level, Mann–Whitney text).

### DISCUSSION

In a previous report (1977) the authors showed that inoculation of live malignant ascites cells into the caecal lumen produces a protective effect against a secondary i.p. challenge with the same tumour cells. The protection could be transferred to untreated animals by sensitized spleen cells. To obtain such an immunization, the ascites cells from the caecal lumen must have invaded the intestinal epithelium and established contact with lymphoid tissue.

One of the basic characteristics of malignant tumour cells is, however, their ability to infiltrate or invade surrounding normal tissues. That the malignant ascites cells used for this work have such an ability seems likely, since an i.p. ascites tumour was developed in C3H mice with impaired immune capacity after inoculation of live ascites cells into the colonic lumen through a small rectal probe, leaving the intestinal epithelium intact (to be published in detail).

In the present study we have dealt with the possibility of enhancing tumour growth by prior immunization *via* the intestine.

An important finding was that 2

inoculations of mammary carcinoma cells into the caecal lumen enhanced the growth of a later s.c. challenge with the same cells in syngeneic C3H mice.

That enhancement induced in this way could be transferred by serum to untreated animals indicates that we are dealing with immunological enhancement as originally defined by Kaliss (1958). The ability of spleen cells to transfer enhancement does not contradict the definition of the phenomenon in question, since specific antibodies could be determined in serum from animals receiving sensitized spleen cells, indicating that some spleen cells are primed antibody-producing cells (to be published).

Inoculated s.c., the mammary tumour cells caused the opposite immune reaction, since the growth of a later transplant was inhibited.

That at least 2 different immune reactions are inducible by intestinal immunization can be more clearly inferred from our work with the malignant ascites cell.

By immunizing with live ascites cells *via* the intracaecal route a protective immune reaction is obtained and the protective effect can be transferred to untreated animals by spleen cells.

In the present study the same ascites cells were used, but the cells were frozen and thawed 5 times before caecal inoculation. By this change (from live to frozen-thawed cells) the very opposite immune reaction appeared. The i.p. tumour grew faster in mice immunized twice with frozen-thawed cells than in non-immunized mice.

An s.c. immunization conferred protection whether live cells or frozen-thawed cells were used. It seems unlikely that an alteration of membrane antigen by freezing and thawing the ascites cells enhanced tumour growth, since enhancement also could be induced by mitomycin-C-treated cells. More likely is the assumption that the different immune reactions obtained by immunization *via* the intracaecal route are due to induction of immunity in different lymphoid cells whether live or treated cells are used.

Purdom, Ambrose and Klein (1958) found, in sublines of ascites sarcoma cells, a direct correlation between the negative surface charge of the cells, as indicated by migration in an electric field, and the degree of invasiveness exhibited in the animal. The ascites form had the highest electrophoretic mobility, whilst the solid line of the tumour had the lowest.

In the present study, the live ascites cell should then be best able to migrate into the intestinal wall, and might even leave this wall *via* the efferent lymphatics. Frozen-thawed cells and mitomycin-C-treated cells, however, do not have this ability to the same degree. As the distribution of Ig-containing cells is not homogeneous throughout the depth of the intestinal wall, it is rendered probable that the immune response differs according to whether sensitization takes place in the mesenteric lymph node, deep in the intestinal wall or in lymphoid cells placed near the lumen.

According to such hypotheses, enhancement should be induced in the intestinal wall by the most luminally placed components of the immune system.

The mammary tumour cells (MTC) originated as a solid tumour and, according to the findings by Purdom *et al.* (1958), had a lesser ability to migrate than had the live ascites cells. Furthermore, the MTC suspension used in this study was a mixture of live and dead cells. By immunizing with this suspension a balanced, or at least partially balanced response should result. In our experiments, the enhancing effect dominated.

The work carried out at the Fibiger Laboratory was sponsored by the Danish Cancer Society.

### REFERENCES

ATTIA, M. A. M. & WEISS, D. W. (1966) Immunology of Spontaneous Mammary Carcinomas in Mice. *Cancer Res.*, **26**, 1787.

FLEXNER, S. & JOBLING, J. W. (1907) On the Promoting Influence of Heated Tumor Emulsions on Tumor Growth. *Proc. Soc. exp. Biol. Med.*, **4**, 156.

KALISS, N. (1952) Regression or Survival of Tumor Homografts in Mice Pretreated with Injections of Lyophilized Tissues. *Cancer Res.*, **12**, 379.

KALISS, N. (1958) Immunological Enhancement of Tumor Homografts in Mice. A Review. *Cancer Res.*, **18**, 992.

LAURSEN, M. L. & LAURSEN, K. (1977) Immunization by the Intestinal Route of C3H-Mice against C3H-L-Ascites Tumor Cells. *Proc. Soc. exp. Biol. Med.*, **154**, 314.

LUND, H. J. C. (1957) Vaccination of Rats against the Yoshida Ascites Sarcoma with the Formation of Complement-fixing Antibody. *Br. J. Cancer*, **11**, 475.

MÖLLER, G. (1964) Effect on Tumour Growth in Syngeneic Recipients of Antibodies against Tumour-specific Antigens in Methylcholanthrene-induced Mouse Sarcomas. *Nature*, **204**, 846.

PURDOM, L., AMBROSE, E. J. & KLEIN, G. (1958) A Correlation between Electrical Surface Charge and some Biological Characteristics during the Stepwise Progression of a Mouse Sarcoma. *Nature*, **181**, 1586.

RAN, M. & WITZ, I. P. (1972) Tumor-associated Immunoglobulins. Enhancement of Syngeneic Tumors by IgG-2 containing Tumor Eluates. *Int. J. Cancer*, **9**, 242.

SNELL, G. D., CLOUDMAN, A. M., FAILOR, E. & DOUGLASS, P. (1946) Inhibition and Stimulation of Tumor Homoiotransplants by the Previous Injection of Lyophilized Tumor Tissue. *J. natn Cancer Inst.*, **6**, 303.

SNELL, G. D. (1970) Immunologic Enhancement. *Surg. Gynec., Obstet.*, **130**, 1109.

TAKASUGI, M. & HILDEMANN, W. H. (1969) Lymphocyte–antibody Interactions in Immunological Enhancement. *Transpl. Proc.*, **1**, 530.